# Supplementary material for: HPLC Analysis and In Vivo Renoprotective Evaluation of Hydroalcoholic Extract of Cucumis melo Seeds in Gentamicin-Induced Renal Damage
Source: Medicina (Kaunas). 2019 Apr 15;55(4):107. doi: 10.3390/medicina55040107 (PMC6524020; doi:10.3390/medicina55040107)
Supplement: Supplementary file 1 [file medicina-55-00107-s001.pdf]

SUPPLEMETARY MATERIAL

Materials and Methods

Different tests for the identification of terpenoids, alkaloids, saponins, tannins, flavonoids and anthraquinone in *CMHE* were performed following reported methods [1].

2.3. Preliminary Qualitative Phytochemical Screening

2.3.1. Glycosides Screening

1.2g of *CMHE* was hydrolyzed by 10 ml of 1% HCl solution and neutralized with10% of NaOH solution follwed by addition of few drops of Fehling's A and B solutions. Presence of glycoside was indicated by formation of red precipitate.

2.3.2. Alkaloidal Screening

200 mg of *CMHE* was heated on a boiling water bath with 5mL of 2N HCl. Cooled mixture was then filtered and divided in to two equal portions. One portion was treated with few drops of Mayer's reagent and other with Dragendeoff's reagent. Presence of alkaloids was indicated by turbidity of resulting precipitates.

2.3.3. Tannins Screening

Approximately 200 mg of *CMHE* was mixed with10 ml of distilled water and the heated on water bath. Later it was filtered and 5% (w /v) solution of ferric chloride was added in the filtrate. Presence of tannins was indicated by formation of dark green solution.

2.3.4. Saponins Screening

Approximately 200 mg of *CMHE* was shaken with 5ml of distilled in test tube and heated on water bath to boil. Presence of saponins was indicated by formation of strong and stable foam.

2.3.5. Flavonoids Screening

Approximately 200 mg of *CMHE* was dissolved in diluted NaOH and 1M of HCl (5ml each). Presence of flavonoids was indicated as yellow solution turned colourless.

2.3.5. Terpenoids Screening

200 mg of *CMHE* was mixed carefully with 2ml of $CHCl_3$ and 3 ml of concentrated H_2S0_4. Presence of terpenes was indicated by formation of reddish brown coloration of the solution at

interface.

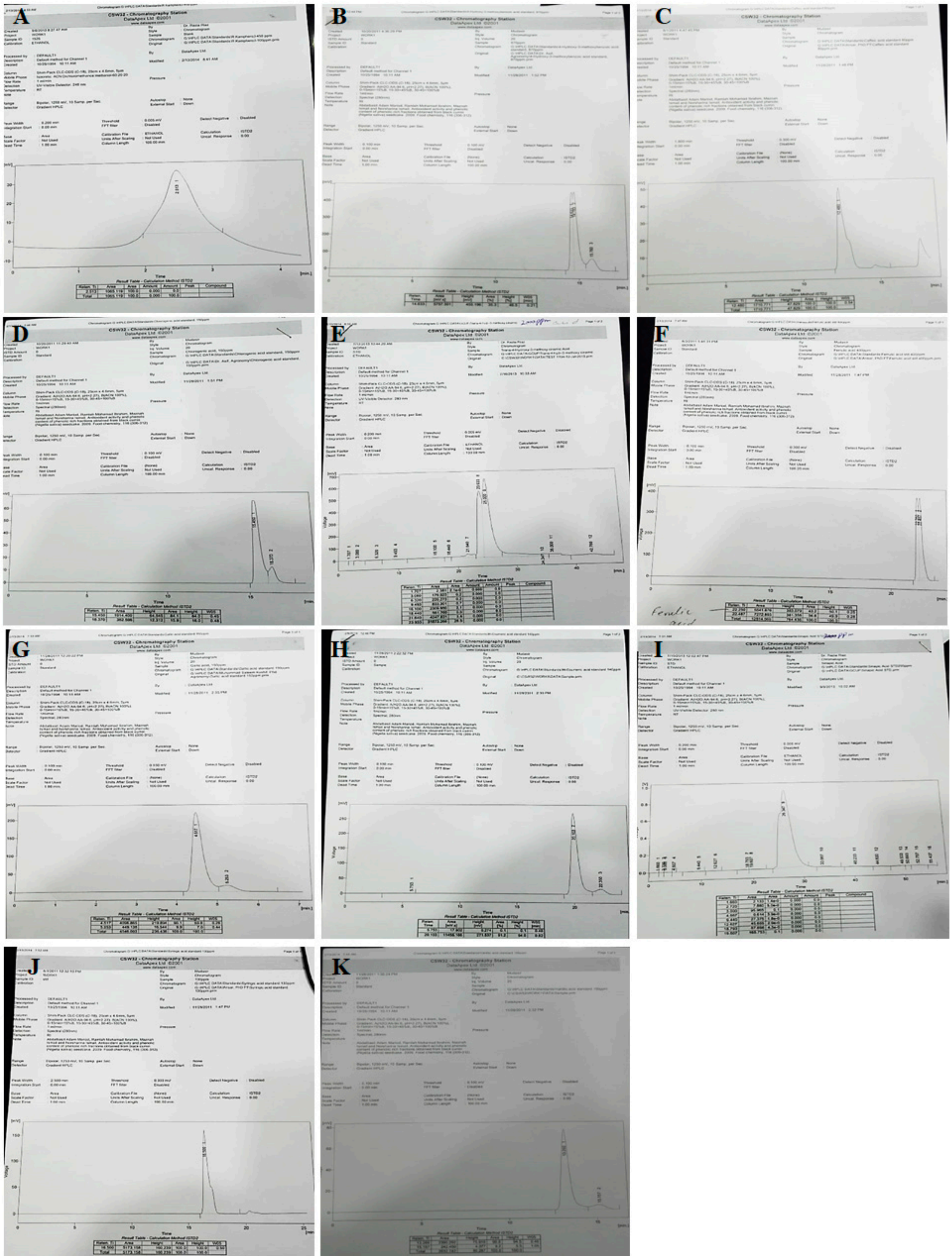

Figure S1. Standard curves of reference compounds. Where A = blank, B = benzoic acid, C = caffeic acid, D = chlorogenic acid, E = cinnamic acid, F = ferulic acid, G = gallic acid, H = coumaric acid, I = sinapic acid, J= syringic acid and K = vanillic acid.

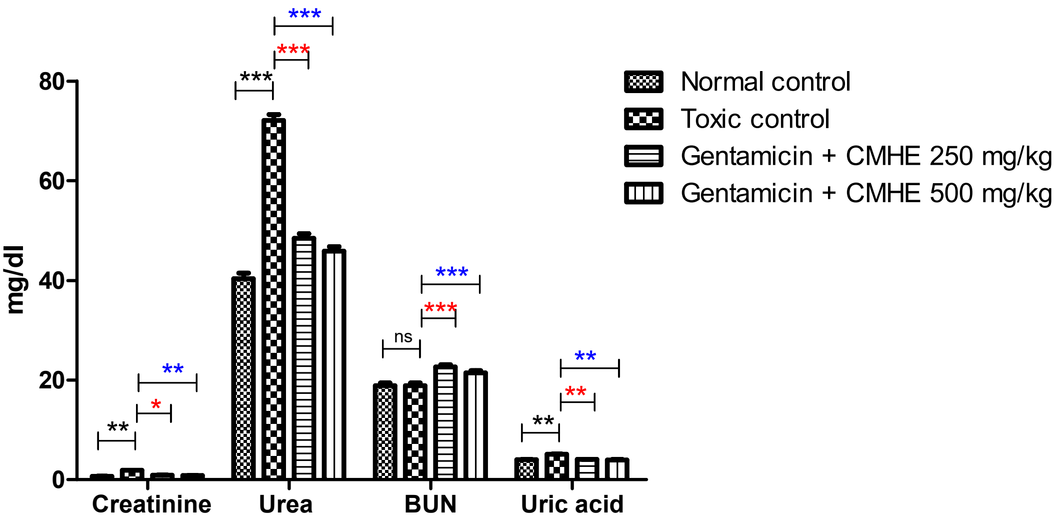

Figure 2. Graphical representation of biochemical parameters. Expressed as mean ± S.D. $n = 6$, where, black asterisks = normal control vs. toxic control; red asterisks = toxic control vs. gentamicin + CMHE 250 mg/kg; blue asterisks = toxic control vs. gentamicin + CMHE 500 mg/kg. * $p < 0.05$, ** $p < 0.01$, *** $p < 0.001$.

References

1. Tadesse, G. Reneela, P. Dekebo, A. Isolation and characterization of natural products from *Helinus mystachnus* (Rhamnaceae). *J. Chem. Pharm. Res.* **2012**, *4*, 1756–1762.
